# Preoperative MRI for predicting pathological changes associated with surgical difficulty during laparoscopic cholecystectomy for acute cholecystitis

**DOI:** 10.1002/bjs5.50344

**Published:** 2020-09-07

**Authors:** K. Omiya, K. Hiramatsu, T. Kato, Y. Shibata, M. Yoshihara, T. Aoba, A. Arimoto, A. Ito

**Affiliations:** ^1^ Department of General Surgery Toyohashi Municipal Hospital 50 Hakken‐Nishi, Aotake‐cho, Toyohashi City Aichi Prefecture 440‐8570 Japan

## Abstract

**Background:**

Severe inflammation with necrosis and fibrosis of the gallbladder in acute cholecystitis increases operative difficulty during laparoscopic cholecystectomy. This study aimed to assess the use of preoperative MRI in predicting pathological changes of the gallbladder associated with surgical difficulty.

**Methods:**

Patients who underwent both preoperative MRI and early cholecystectomy for acute cholecystitis between 2012 and 2018 were identified retrospectively. On the basis of the layered pattern of the gallbladder wall on MRI, patients were classified into three groups: high signal intensity (HSI), intermediate signal intensity (ISI), and low signal intensity (LSI). The endpoint was the presence of pathological changes of the gallbladder associated with surgical difficulty, such as necrosis, abscess formation and fibrosis.

**Results:**

Of 229 eligible patients, pathological changes associated with surgical difficulty were found in 17 (27 per cent) of 62 patients in the HSI group, 84 (85 per cent) of 99 patients in the ISI group, and 66 (97 per cent) of 68 patients in the LSI group (*P* < 0·001). For detecting these changes, intermediate to low signal intensity of the gallbladder wall had a sensitivity of 90 (95 per cent c.i. 84 to 94) per cent, specificity of 73 (60 to 83) per cent and accuracy of 85 (80 to 90) per cent.

**Conclusion:**

Preoperative MRI predicted pathological changes associated with surgical difficulty during laparoscopic cholecystectomy for acute cholecystitis.

## Graphical abstract

Variations in the layered pattern of the gallbladder wall on MRI were significantly associated with fibrosis and necrosis of the gallbladder wall in acute cholecystitis. Intermediate to low signal intensity of the gallbladder wall on MRI had a sensitivity of 90 per cent and a specificity of 73 per cent for predicting pathological changes in the gallbladder wall associated with surgical difficulty during laparoscopic cholecystectomy for acute cholecystitis. The results indicate that MRI could be a novel method of predicting surgical difficulty in laparoscopic cholecystectomy.

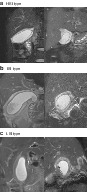

MRI could possilby predict surgical difficulty of cholecystectomy.

## Abstract (es)

**Antecedentes:**

La inflamación grave con necrosis y fibrosis de la vesícula biliar en la colecistitis aguda aumenta la dificultad quirúrgica durante la colecistectomía laparoscópica. Este estudio tuvo como objetivo evaluar el uso de la resonancia magnética preoperatoria (*magnetic resonance imaging*, MRI) para predecir los cambios patológicos de la vesícula biliar asociados con la dificultad quirúrgica.

**Métodos:**

Los pacientes que se sometieron tanto a MRI preoperatoria como a colecistectomía precoz por colecistitis aguda entre 2012 y 2018 fueron identificados retrospectivamente. En base a la distribución en capas de la pared de la vesícula biliar en la MRI, los pacientes se clasificaron en tres grupos: (1) intensidad de señal alta (*high signal intensity*, HSI), (2) intensidad de señal intermedia (*intermediate signal intensity*, ISI) y (3) intensidad de señal baja (*low signal intensity*, LSI). El objetivo final fue la presencia de cambios patológicos en la vesícula biliar asociados con la dificultad quirúrgica, tales como necrosis, formación de abscesos y fibrosis.

**Resultados:**

De los 229 pacientes elegibles, se documentaron cambios patológicos asociados con dificultad quirúrgica en 17 (27,4%) de 62 pacientes en el grupo HSI, 84 (84,8%) de 99 pacientes en el grupo ISI y 66 (97,1%) de 68 pacientes en el grupo LSI (*P* < 0,001). Para detectar estos cambios, la intensidad de señal de intermedia a baja de la pared de la vesícula biliar tuvo una sensibilidad del 89,8% (i.c. del 95% 84,2%‐94,0%), una especificidad del 72.6% (i.c. del 95% 59,8%‐83,1%) y una precisión del 85,2% (i.c. del 95% 79,9%‐89,5%).

**Conclusión:**

La MRI preoperatoria predijo los cambios patológicos asociados con la dificultad quirúrgica durante la colecistectomía laparoscópica por colecistitis aguda.

## Introduction

Surgical difficulty during laparoscopic cholecystectomy (LC) for acute cholecystitis (AC) differs, depending on the degree of inflammation and fibrosis of the gallbladder^1^. Severe local inflammation and fibrosis of the gallbladder wall increase both operative difficulty and frequency of intraoperative complications during LC^1^, ^2^. The Tokyo Guidelines from 2018^1^ proposed that intraoperative findings such as necrosis, abscess formation and fibrosis of the gallbladder are considered to be novel, objective and direct indicators for measuring surgical difficulty in LC for AC. Indicators such as open conversion rate and duration of surgery are not considered appropriate because they depend substantially on surgeons' skills and experiences^3^, ^4^. However, in clinical practice, preoperative predictions of these pathological changes in the gallbladder wall are also crucial in deciding a treatment strategy to reduce severe complications.

These histopathological changes of the gallbladder wall in AC develop with time within the following stages: first stage, oedematous cholecystitis (2–4 days); second stage, necrotizing cholecystitis (3–5 days); third stage, suppurative cholecystitis (7–10 days); and fourth stage, chronic cholecystitis (repeated occurrence of cholecystitis)^5^. The second and third stages comprise gangrenous changes, and the third and fourth stages comprise fibrosis of the gallbladder wall^5^.

However, it is difficult to determine precisely the time that has passed since disease onset^6^. In addition, some cases of AC occur as a result of exacerbations of chronic cholecystitis^6^. Therefore, it is difficult to predict the pathological condition of the gallbladder in AC, and surgeons often discover unpredictable severe necrosis and fibrosis of the gallbladder wall during early surgery, sometimes even within 48 h of disease onset. Although many studies have tried to identify indicators of surgical difficulty in LC, only a few have focused on preoperative assessment to predict pathological changes of the gallbladder wall associated with increased surgical difficulty.

Some studies have reported that the gallbladder wall showed various signal intensities and layered patterns on MRI; the MRI findings correlated well with the pathological findings and were useful for diagnosis of gallbladder disease, such as AC, gangrenous cholecystitis, chronic cholecystitis and gallbladder carcinoma^7^, ^8^, ^9^, ^10^. The present authors have assumed that layered patterns of the gallbladder wall in AC before surgery are also associated with pathological changes associated with surgical difficulty, such as necrosis, abscess formation and fibrosis.

This study aimed to assess the usefulness of preoperative non‐contrast‐enhanced MRI to predict the pathological changes of the gallbladder wall associated with increased surgical difficulty during early cholecystectomy for AC.

## Methods

An institutional surgery database was searched to identify patients who had undergone cholecystectomy at Toyohashi Municipal Hospital in Japan from January 2012 to December 2018. Patient information was collected retrospectively from the electronic medical records. Eligibility criteria were: patients with AC diagnosed clinically according to the Tokyo Guidelines of 2007^11^, 2013^12^ and 2018^13^, who underwent cholecystectomy 7 days or less after disease onset (early cholecystectomy), and had MRI and magnetic resonance cholangiopancreatography (MRCP) 24 h or less before surgery. Exclusion criteria were: gallbladder wall thickness less than 3 mm on MRI, and clinical suspicion of gallbladder cancer. The ethics committee of Toyohashi Municipal Hospital approved the study protocol.

### MRI and MRCP procedure

Non‐contrast‐enhanced MRI and MRCP on a 3‐T superconducting instrument (MAGNETOM Skyra; Siemens, Erlangen, Germany) were performed routinely to assess the presence of common bile duct stones and abnormal anatomical variations in the bile duct before surgery. After initial T1‐weighted images, HASTE (Half‐Fourier‐Acquired Single‐shot Turbo spin Echo) T2‐weighted images sequence were applied in the axial, coronal, and oblique sagittal planes. The oblique sagittal plane was applied parallel to the course of the common bile duct, as demonstrated on initial coronal scout views. Images were acquired in each imaging plane. MRCP was performed using a HASTE sequence. The following parameters were used: prospective acquisition correction technique; repetition time, ∞; echo time, 86 ms; thickness, 3 mm; gap, 0 mm; flip angle, 130°; matrix size, 256 × 256; phase partial Fourier 4/8; field of view, 300 × 300 mm; and fat saturation (spectral‐attenuated inversion recovery). Data from the MRCP and original HASTE MRI of each patient were routinely stored in the electronic medical records.

### Interpretation of MRI

**Fig. 1:**
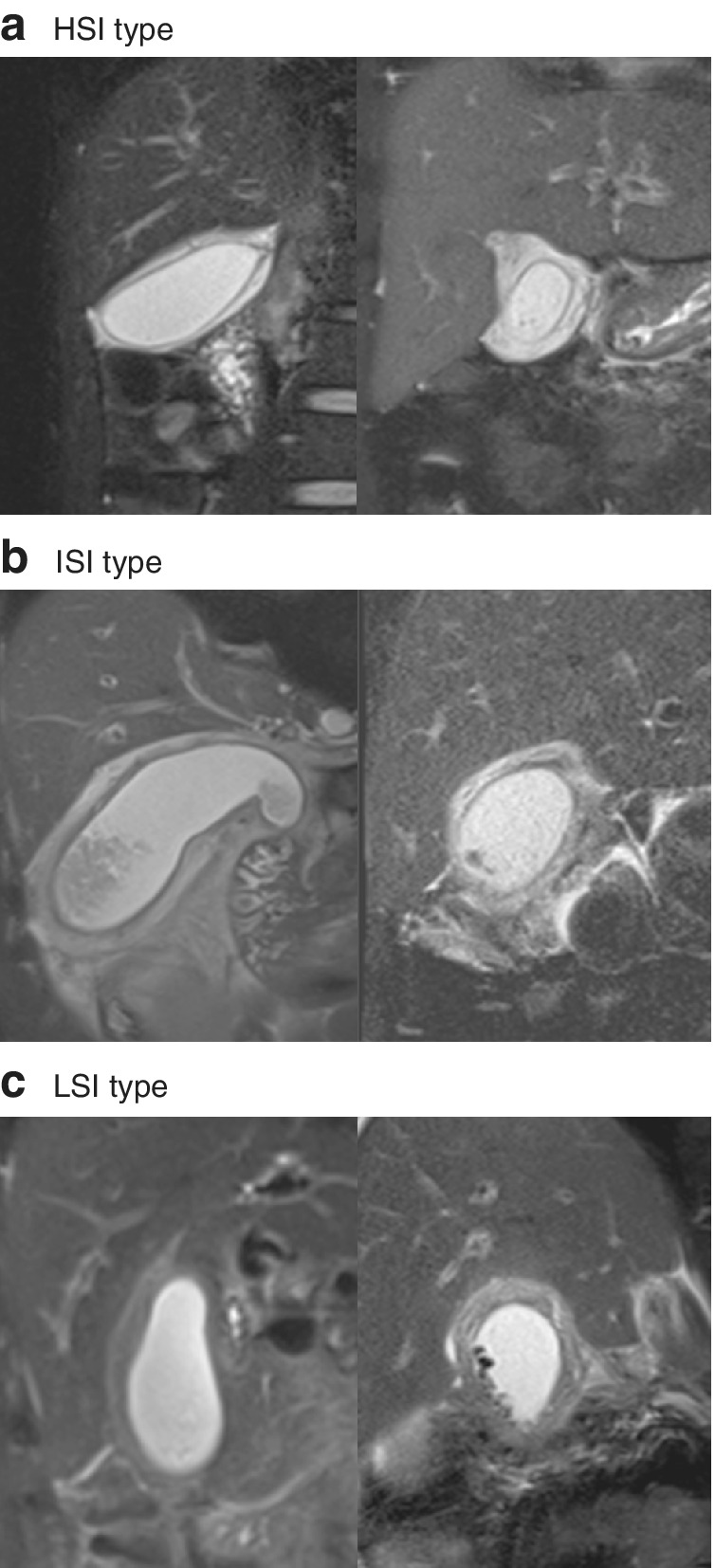
Layered pattern of the gallbladder wall **a** High signal intensity (HSI) type: thin inner layer with low signal intensity (LSI), thickened outer layer with HSI, and well defined margin. **b** Intermediate signal intensity (IS) type: partially thickened inner layer and ill‐defined margin. **c** LSI type: diffusely thickened LSI layer.

The previously obtained preoperative MRI and MRCP data for all patients were assessed retrospectively and independently by two surgeons blinded to the clinical information and type of surgery but aware of the presence of cholecystitis. HASTE T2‐weighted images captured during MRCP were assessed, and gallbladder wall thickness was measured from the section showing the thickest part of the wall. The layered pattern of the thickened wall was classified into three groups: a high signal intensity (HSI) group (two layers with a discrete margin composed of a thin inner layer (3 mm or less) with low signal and a relatively thick outer layer with high signal); an intermediate signal intensity (ISI) group (two layers with a partially ill‐defined margin composed of a partially thickened inner layer (more than 3 mm) with low signal and an outer layer with high or partially heterogeneous intermediate signal); and a low signal intensity (LSI) group (ill‐defined layers composed of a diffusely thickened inner layer (more than 3 mm) with low signal and an outer layer with intermediate to low signal). Examples of images from the three groups are shown in *Fig*. *1*. Signal intensities were determined by using standardized regions of interest (ROI). The ROI size was similar for all measurements and patients, and varied between 0·03 and 0·06 cm^2^. LSI lesions of the gallbladder wall were judged in comparison with the signal intensity of the renal parenchyma.

### Histopathological examination

Details of histopathological findings in the surgical specimens of each patient were obtained from the pathology reports. These reports were made within 2 weeks of surgery by a staff pathologist who was aware of the presence of cholecystitis and type of surgery but was not informed about the radiological findings.

### Endpoint

The endpoint of this study was the presence of pathological changes predictive of surgical difficulty, such as necrosis, abscess formation or fibrosis. On the basis of the results of the three groups, the appropriate cut‐off point for MRI findings of the gallbladder wall was determined for predicting pathological changes associated with surgical difficulty. Sensitivity, specificity, accuracy, positive likelihood ratio (LR+) and negative likelihood ratio (LR−) were calculated. Subgroup analyses were performed to assess the use of preoperative MRI findings for predicting the pathological condition of the gallbladder wall in patients with AC who had undergone surgery within 48 h of disease onset.

### Statistical analysis

Median (range) values are presented. Fisher's exact test was used to test differences between categorical variables, and the Mann‐Whitney *U* test and the Kruskal–Wallis rank sum test for differences between continuous variables. All *P* values were two‐sided, and associations were considered significant at *P* < 0·050. If there was a significant difference among the three groups, pairwise comparisons for all groups were performed, with *P* values adjusted by the Holm method. All statistical analyses were performed using R version 3.5.2 (The R Foundation for Statistical Computing, Vienna, Austria) and EZR (Saitama Medical Centre, Jichi Medical University, Saitama, Japan), which is a graphical user interface for R.

## Results

**Fig. 2:**
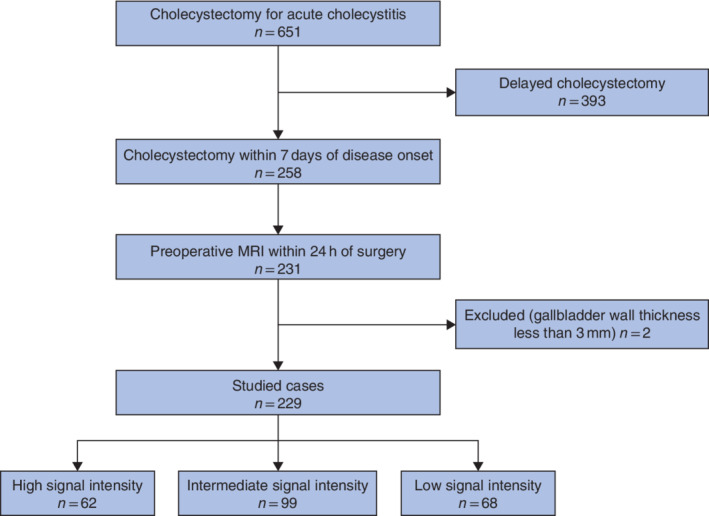
Flow diagram of patient selection

Data were collected for 651 patients who had underwent cholecystectomy after diagnosis of AC. Of these patients, 258 had undergone early cholecystectomy and 393 had had delayed cholecystectomy. Some 231 of the patients who underwent early cholecystectomy had MRI and MRCP within 24 h of surgery. Two patients were excluded owing to gallbladder wall thickness of less than 3 mm on MRI. Ultimately, 62, 99 and 68 patients respectively were selected for the HSI, ISI and LSI groups *(Fig*. *2*).

*Table* *1* shows baseline patient characteristics, preoperative findings and type of operation. Baseline characteristics were similar among the three groups. Gallbladder wall thickness measured on MRI was significantly greater in the ISI and LSI groups than in the HSI group (HSI *versus* ISI, *P* < 0·001; ISI *versus* LSI, *P* = 0·277; LSI *versus* HSI, *P* < 0·001). Preoperative C‐reactive protein (CRP) level was significantly higher in decreasing order of LSI, ISI and HSI in pairwise comparisons (HSI *versus* ISI, *P* < 0·001; ISI *versus* LSI, *P* < 0·001; LSI *versus* HSI, *P* < 0·001). The rate of planned open cholecystectomy was significantly higher in decreasing order of LSI, ISI and HSI (HSI *versus* ISI, *P* = 0·022; ISI *versus* LSI, *P* = 0·022; LSI *versus* HSI, *P* < 0·001).

**Table 1:** Characteristics of the three groups

	HSI group (*n* = 62)	ISI group (*n* = 99)	LSI group (*n* = 68)	*P* ‡
**Baseline characteristics**				
Age (years)*	59 (46–68)	65 (51–73)	67 (57–75)	0·068§
Sex ratio (M : F)	36 : 26	67 : 32	45 : 23	0·439
BMI (kg/m^2^)*	25·0 (21·3–28·1)	24·3 (21·8–27·4)	24·8 (22·4–27·1)	0·878§
ASA physical status				0·037
I	28 (45)	34 (34)	21 (31)	
II	34 (55)	59 (60)	39 (57)	
III	0 (0)	6 (6)	8 (12)	
Diabetes mellitus	11 (18)	14 (14)	15 (22)	0·418
Previous diagnosis of gallstones	17 (27)	34 (34)	22 (32)	0·793
Past gallbladder attack	13 (21)	17 (17)	14 (21)	0·790
Past acute cholecystitis	3 (5)	7 (7)	7 (10)	0·530
**Preoperative findings**				
Body temperature (°C)*	36·7 (36·3–37·2)	37·0 (36·6–37·5)	37·0 (36·6–37·6)	0·029§
Preoperative gallbladder drainage	0 (0)	2 (2)	1 (1)	0·789
Thickness of gallbladder wall on MRI (mm)*	5 (4–7)	7 (6–8)	7 (6–9)	< 0·001§
Gallstones recognized on MRI	53 (85)	83 (84)	53 (78)	0·117
WBC (cells/μl)*	10 135 (8445–13 960)	10 670 (8835–14 615)	12 715 (9470–15 512)	0·054§
CRP (mg/dl)*	0·31 (0·08–1·05)	0·60 (0·14–5·99)	6·51 (1·18–15·14)	< 0·001§
AST (units/l)*	24 (19–37)	24 (18–33)	24 (19–34)	0·721§
ALT (units/l)*	26 (18–39)	23 (17–41)	25 (16–52)	0·519§
ALP (units/l)*	213 (163–283)	223 (184–289)	221 (183–282)	0·442§
Total bilirubin (mg/dl)*	0·7 (0·5–1·0)	1·0 (0·7–1·6)	1·0 (0·7–1·5)	< 0·001§
Severity grade†				0·037
I	60 (97)	86 (87)	57 (84)	
II	2 (3)	13 (13)	11 (16)	
**Surgical details**				
Type of surgery				< 0·001
Laparoscopic	60 (97)	81 (82)	42 (62)	
Open conversion	0 (0)	8 (8)	14 (21)	
Open	2 (3)	10 (10)	12 (18)	
Time from disease onset to surgery (h)				0·114
≤ 48	55 (89)	78 (79)	51 (75)	
> 48	7 (11)	21 (21)	17 (25)	

Values in parentheses are percentages unless indicated otherwise;

* values are median (range).

† Assessed according to Tokyo Guidelines of 2007, 2013 and 2018. HSI, high signal intensity; ISI, intermediate signal intensity; LSI, low signal intensity; WBC, white blood cell; CRP, C‐reactive protein; AST, aspartate aminotransferase; ALT, alanine aminotransferase; ALP, alkaline phosphatase.

‡ Fisher's exact test, except

§ Kruskal–Wallis test.

### Outcomes

Pathological gallbladder changes associated with surgical difficulty were identified in 27 per cent of patients in the HSI group, 85 per cent of patients in the ISI group, and 97 per cent of those in the LSI group (*P* < 0·001) (*Table* *2*). In the pairwise comparisons, the proportion of patients with any pathological change associated with surgical difficulty was significantly higher in decreasing order of LSI, ISI and HSI (*Table* *2*).

**Table 2:** Pathological outcomes in the three groups

	HSI group (*n* = 62)	ISI group (*n* = 99)	LSI group (*n* = 68)	*P* *
**Pathological change associated with surgical difficulty**	17 (27)	84 (85)	66 (97)	< 0·001†
**Details of pathological change**				
Necrosis	9 (15)	51 (52)	49 (72)	< 0·001†
Abscess formation	2 (3)	8 (8)	9 (13)	0·126
Fibrosis	10 (16)	50 (51)	37 (54)	< 0·001‡

Values in parentheses are percentages. HSI, high signal intensity; ISI, intermediate signal intensity; LSI, low signal intensity.

* Fisher's exact test.

† In pairwise comparisons: *P* < 0·001 (HSI *versus* ISI), *P* = 0·010 (ISI *versus* LSI) and *P* < 0·001 (LSI *versus* HSI);

‡ in pairwise comparisons: *P* < 0·001 (HSI *versus* ISI), *P* = 0·640 (ISI *versus* LSI) and *P* < 0·001 (LSI *versus* HSI) (Fisher's exact test with adjustment by Holm method).

With regard to the types of pathological change, necrosis in the gallbladder wall was detected in 15 per cent of patients in the HSI group, 52 per cent of patients in the ISI group, and 72 per cent of those in the LSI group. In the pairwise comparisons, the proportion of patients with necrosis of the gallbladder was significantly higher in decreasing order of LSI, ISI and HSI (*Table* *2*). Fibrosis in the gallbladder wall was detected in 16 per cent of the HSI group, 51 per cent of the ISI group, and 54 per cent of the LSI group. In the pairwise comparisons, the proportion of patients with fibrosis of the gallbladder was significantly higher in the ISI and LSI groups than in the HSI group (*Table* *2*). Abscess formation in the gallbladder wall was found in 3, 8 and 13 per cent of patients in the HSI, ISI and LSI group respectively, but the differences were not significant (*Table* *2*).

### Use of MRI to predict gallbladder wall changes associated with surgical difficulty

As the outcomes were relatively similar between ISI and LSI groups, intermediate to low signal intensity in the gallbladder wall was defined as positive and high signal intensity as negative. The sensitivity, specificity, accuracy, LR+ and LR− of MRI for predicting pathological changes associated with surgical difficulty were 90 (95 per cent c.i. 84 to 94) per cent, 73 (60 to 83) per cent, 85 (80 to 90) per cent, 3·28 (95 per cent c.i. 2·18 to 4·93) and 0·14 (0·09 to 0·23) respectively. Because preoperative CRP concentration was significantly higher in the decreasing order of LSI, ISI and HSI (*Tables* *1* and *3*), an additional analysis was performed for the utility of CRP level in predicting pathological change; this was later compared with MRI scans. The resulting Mann–Whitney *U* test highlighted a significant difference between preoperative CRP level and pathological changes. However, as with all cut‐off points, the accuracy, LR+ and LR− values of CRP for predicting pathological changes associated with surgical difficulty were inferior to those of MRI.

**Table 3:** Characteristics of the subgroups of patients undergoing surgery within 48 h of disease onset

	HSI group (*n* = 55)	ISI group (*n* = 78)	LSI group (*n* = 51)	*P* ‡
**Baseline characteristics**				
Age (years)*	60 (49–69)	65 (51–71)	67 (59–75)	0·129§
Sex ratio (M : F)	32 : 23	53 : 25	31 : 20	0·439
BMI (kg/m^2^)*	25·0 (21·3–28·1)	24·3 (21·8–27·4)	24·8 (22·4–27·1)	0·484§
ASA physical status				0·026
I	26 (47)	26 (33)	17 (33)	
II	29 (53)	48 (62)	27 (53)	
III	0 (0)	4 (5)	7 (14)	
Diabetes mellitus	10 (18)	11 (14)	13 (25)	0·262
Previous diagnosis of gallstones	14 (25)	27 (35)	18 (35)	0·471
Past gallbladder attack	11 (20)	14 (18)	11 (22)	0·891
Past acute cholecystitis	2 (4)	4 (5)	6 (12)	0·244
**Preoperative findings**				
Body temperature (°C)*	36·6 (36·2–37·2)	36·9 (36·3–37·5)	36·9 (36·5–37·7)	0·038§
Preoperative gallbladder drainage	0 (0)	1 (1·3)	0 (0)	1·000
Thickness of gallbladder wall on MRI (mm)*	5 (4–7)	7 (6–8)	7 (6–9)	< 0·001§
Gallstones recognized on MRI	47 (85)	67 (86)	42 (82)	0·869
WBC (cells/μl)*	11 260 (8850–14 140)	10 595 (8873–14 060)	12 690 (9440–15 835)	0·141§
CRP (mg/dl)*	0·25 (0·08–0·85)	0·52 (0·12–5·12)	3·18 (0·47–11·39)	< 0·001§
AST (units/l)*	24 (19–34)	25 (18–33)	25 (19–42)	0·972§
ALT (units/l)*	25 (18–38)	23 (17–43)	25 (16–54)	0·919§
ALP (units/l)*	206 (161–272)	222 (182–288)	217 (174–285)	0·240§
Total bilirubin (mg/dl)*	0·6 (0·2–0·9)	1·0 (0·7–1·6)	1·0 (0·7–1·5)	< 0·001§
Severity grade†				0·049
I	53 (96)	68 (87)	42 (82)	
II	2 (4)	10 (13)	9 (18)	
**Type of surgery**				< 0·001
Laparoscopic	55 (100)	66 (85)	33 (65)	
Open conversion	0 (0)	6 (8)	11 (22)	
Open	0 (0)	6 (8)	7 (14)	

Values in parentheses are percentages unless indicated otherwise;

* values are median (range).

† Assessed according to Tokyo Guidelines of 2007, 2013 and 2018. HSI, high signal intensity; ISI, intermediate signal intensity; LSI, low signal intensity; WBC, white blood cell; CRP, C‐reactive protein; AST, aspartate aminotransferase; ALT, alanine aminotransferase; ALP, alkaline phosphatase.

‡ Fisher's exact test, except

§ Kruskal–Wallis test.

### Subgroup analysis

A total of 184 patients who had surgery within 48 h of disease onset were eligible for subgroup analysis. Pathological changes associated with surgical difficulty, such as necrosis, abscess formation and fibrosis, were detected in 130 (70·7 per cent) of these patients: 55 in the HSI group, 78 in the ISI group and 51 in the LSI group. Subgroup results are shown in *Table* *4*. For each group, the proportion of patients with pathological changes associated with surgical difficulty was similar to that in analysis of the whole cohort.

**Table 4:** Pathological outcomes in the subgroup of patients undergoing surgery within 48 h of disease onset

	HSI group (*n* = 55)	ISI group (*n* = 78)	LSI group (*n* = 51)	*P* *
**Pathological change associated with surgical difficulty**	14 (25)	66 (85)	50 (98)	< 0·001†
**Details of pathological change**				
Necrosis	8 (15)	36 (46)	37 (73)	< 0·001‡
Abscess formation	2 (4)	5 (6)	7 (14)	0·149
Fibrosis	7 (13)	40 (51)	26 (51)	< 0·001§

Values in parentheses are percentages. HSI, high signal intensity; ISI, intermediate signal intensity; LSI, low signal intensity.

* Fisher's exact test.

† In pairwise comparisons: *P* < 0·001 (HSI *versus* ISI), *P* = 0·015 (ISI *versus* LSI) and *P* < 0·001 (LSI *versus* HSI);

‡ in pairwise comparisons: *P* < 0·001 (HSI *versus* ISI), *P* = 0·004 (ISI *versus* LSI) and *P* < 0·001 (LSI *versus* HSI);

§ in pairwise comparison: *P* < 0·001 (HSI *versus* ISI), *P* = 1·000 (ISI *versus* LSI) and *P* < 0·001 (LSI *versus* HSI) (Fisher's exact test with adjustment by Holm method).

The sensitivity, specificity, accuracy, LR+ and LR− of MRI in identifying pathological changes associated with surgical difficulty of gallbladder wall in patients with AC who had surgery within 48 h of disease onset were 89 (95 per cent c.i. 83 to 94) per cent, 76 (62 to 87) per cent, 85 (79 to 90) per cent), 3·71 (95 per cent c.i. 2·30 to 5·98) and 0·14 (0·09 to 0·24) respectively.

## Discussion

There were three major findings from this single‐centre retrospective study. First, variations in the layered pattern of the gallbladder wall on non‐contrast‐enhanced MRI classified by the study criteria were significantly associated with fibrosis and necrosis of the gallbladder wall in patients with AC. Second, intermediate to low signal intensity of the gallbladder wall had a sensitivity of 90 per cent and a specificity of 73 per cent for predicting pathological changes in the gallbladder wall associated with surgical difficulty during LC in AC. Third, even in patients who had early cholecystectomy within 48 h of disease onset, 70·7 per cent had pathological changes associated with surgical difficulty, and MRI findings were useful in predicting such changes. These results indicate that MRI is a promising method for predicting surgical difficulty for LC.

Many previous studies^14^, ^15^, ^16^, ^17^, ^18^, ^19^, ^20^ have reported predictors of surgical difficulty during LC for AC, such as preoperative radiological findings (non‐visualized gallbladder on preoperative cholangiography, cystic duct length, gallbladder wall thickening, incarcerated stones in the gallbladder neck, fluid retention around the gallbladder), duration of raised CRP, white blood cell count, low albumin, high bilirubin, diabetes and male sex. These studies included factors such as open conversion rate and duration of surgery as indicators of surgical difficulty. However, some studies^3^, ^21^ have indicated that the criteria for open conversion vary among surgeons and that the operating time is highly dependent on the skills and experience of the operator. Based on a Delphi consensus amongst a large number of surgeons of varying nationalities, Iwashita and colleagues^3^, ^4^ reported that intraoperative findings that have a substantial effect on surgical difficulty during LC, such as necrosis, abscess formation and fibrosis of the gallbladder wall, may be novel indicators of surgical difficulty. The Tokyo Guidelines 2018 recommended the use of these intraoperative findings as objective, direct indicators that are capable of measuring surgical difficulty. As a next step, the present authors focused here on the preoperative prediction of these pathological conditions of the gallbladder wall in patients with AC.

MRCP is a non‐invasive technique that has been used previously in the assessment of bile duct abnormality. The HASTE sequence captured for MRCP has already been shown to be a technique for assessing pathology of the gallbladder wall. Jung *et al*.^7^ reported that the thickened gallbladder wall on HASTE MRI had two layers: a low‐signal inner layer and a high‐signal outer layer. Pathologically, the inner layer corresponded to the mucosa and muscular layer, and the outer layer corresponded to serosal oedema. These researchers also reported that, in some cases, thickening of the low‐signal layer with an ill‐defined margin corresponded to sloughed mucosa or haemorrhagic necrosis, and the ISI area of the outer layer corresponded to prominent fibrosis in the muscular layer and serosa. The results of the present study may be radiologically and pathologically compatible.

In general, necrosis and fibrosis of the gallbladder wall occur approximately 3 and 7 days, respectively, after the onset of AC^5^. Some studies^22^, ^23^, ^24^, ^25^, ^26^ assessing the appropriate timing for surgery have reported that early LC within 72 h of disease onset is relatively safe, and the Tokyo Guidelines 2013 previously recommended early cholecystectomy within 72 h of the onset of symptoms^27^. However, in the present study, approximately 70 per cent of patients who had early surgery within 48 h after onset of AC already had either fibrosis, necrosis or abscess formation of the gallbladder wall. This finding might be because, in some cases, AC had begun some days before the patients noticed their symptoms or AC occurred in the mechanism of acute exacerbations of chronic cholecystitis. The present results indicate that MRI findings might also be useful for predicting such clinically unpredictable pathological conditions.

Several limitations of the present study should be acknowledged. First, this was a retrospective analysis. As the outcomes were assessed on the basis of the pathological report from a staff pathologist before the study protocol was devised, mild pathological changes might not have been recorded. In addition, the study included only patients who had undergone MRI within 24 h of surgery. Gangrenous change of the gallbladder wall in AC may progress day by day. Although some extension of the time between MRI and surgery might be acceptable, care should be taken when extrapolating the present results to patients under other circumstances. Furthermore, during the 2012–2018 study period, most patients who were diagnosed as having AC more than 72 h from disease onset underwent delayed surgery in the authors' hospital, because the Tokyo Guidelines 2013 recommended early surgery only for patients with disease onset of 72 h or less^27^. Further validation is necessary before patients diagnosed as having AC 72 h after onset can be assessed.
